# Modelling the relationship between obesity and mental health in children and adolescents: findings from the Health Survey for England 2007

**DOI:** 10.1186/1753-2000-5-31

**Published:** 2011-10-07

**Authors:** Paul A Tiffin, Bronia Arnott, Helen J Moore, Carolyn D Summerbell

**Affiliations:** 1 School of Medicine and Health, Wolfson Research Institute, Durham University Queen's Campus, University Boulevard, Stockton-on-Tees, TS17 6BH, UK; 2 Child Development Unit, Wolfson Research Institute, Durham University Queen's Campus, University Boulevard, Stockton-on-Tees, TS17 6BH, UK

**Keywords:** Obesity, Children, Adolescents, Mental Health, Statistical Modelling

## Abstract

A number of studies have reported significant associations between obesity and poor psychological wellbeing in children but findings have been inconsistent. Methods: This study utilised data from 3,898 children aged 5-16 years obtained from the Health Survey for England 2007. Information was available on Body Mass Index (BMI), parental ratings of child emotional and behavioural health (Strengths and Difficulties Questionnaire), self-reported physical activity levels and sociodemographic variables. A multilevel modelling approach was used to allow for the clustering of children within households. Results: Curvilinear relationships between both internalising (emotional) and externalising (behavioural) symptoms and adjusted BMI were observed. After adjusting for potential confounders the relationships between obesity and psychological adjustment (reported externalising and internalising symptoms) remained statistically significant. Being overweight, rather than obese, had no impact on overall reported mental health. 17% of children with obesity were above the suggested screening threshold for emotional problems, compared to 9% of non-obese children. Allowing for clustering and potential confounding variables children classified as obese had an odds ratio (OR) of 2.13 (95% CI 1.39 to 3.26) for being above the screening threshold for an emotional disorder compared to non-obese young people. No cross-level interactions between household income and the relationships between obesity and internalising or externalising symptoms were observed. Conclusions: In this large, representative, UK-based community sample a curvilinear association with emotional wellbeing was observed for adjusted BMI suggesting the possibility of a threshold effect. Further research could focus on exploring causal relationships and developing targeted interventions.

## Background

Childhood obesity is a serious health problem in the Western world with evidence of continued high rates [1,2]. Moreover, excess adiposity in children tracks throughout adulthood [3] and is linked to serious physical health risks [4]. Thus, a continued paediatric obesity epidemic will be associated with increased long-term health and social care costs and decreased productivity at a time of global economic downturn [5]. Rates of mental health problems in young people are also high, and increasing, with around one in ten children aged 5-16 years having a diagnosable condition [6,7]. Like obesity, mental ill health has been identified as a major cause of persistent disability with attendant economic implications [8].

Obesity has been shown to be associated with poor mental health in studies of working-age adults [9,10] with most research focussed on depression. A meta-analysis pooling the results of 17 cross-sectional studies concluded that the association between obesity and depression was highly statistically significant and possibly varied by gender [11]. There are many plausible reasons why excess adiposity may be associated with poor psychological adjustment. These include: the impact of obesity on self-esteem and social confidence; the direct effect of hormonal and metabolic changes on brain function [12,13]; the result of changes in dietary behaviour and physical activity levels that can be a consequence of depressed mood [14] or; weight gain secondary to the use of psychiatric medications [15]. In adults, the causal mechanism underlying the association between depression and obesity appears to be bidirectional: a meta-analysis using the findings of 15 longitudinal studies of predominantly working-age adults concluded that the Odds Ratio (OR) of being obese at follow-up was 1.58 (95%CI 1.33-1.87). Conversely the ORs of being depressed at follow-up was 1.55 (95% CI 1.22-1.98) if obese and 1.27 (95% CI 1.07 -1.51) if overweight at initial evaluation [16]. Interestingly, the meta-analysis included four studies where the average age at baseline assessment was below 18 years (with follow-up in adulthood). In these cases there was no observed association between overweight at baseline and risk of depression at follow-up. Nevertheless, an increased risk of depression at follow-up was observed with initial obesity. Such studies also provide evidence that those experiencing depression during adolescence may be at increased risk of obesity in adulthood [17].

However, previous cross-sectional work investigating the possible association between obesity and psychopathology among community-based samples of children have reported mixed findings. A number of surveys have reported a statistically significant and independent relationship between aspects of poor psychological adjustment and increased Body Mass Index (BMI) in children, though the nature and strength of these associations have varied [18-22]. For example, one Swedish survey reported a significant association between depression and obesity in a sample of 4,703 15-17 year olds [18]. There have also been some studies that have reported a link between behavioural problems and weight in children [18,23]. For instance, early findings from the UK-based Millenium cohort study also highlight a gender-specific association between obesity and behavioural difficulties in children under five years [22]. Few robust longitudinal data have been available concerning mental health and weight during childhood and adolescence. However, one recent systematic review concluded that, despite inconsistencies in methodology and sample characteristics, the most consistent psychological precursor to obesity reported in under 18s was low self-esteem [24]. Other studies have not observed a relationship between childhood adiposity and psychopathology once potentially confounding sociodemographic variables such as ethnicity, age, gender and socioeconomic status have been controlled for [25-27].

Low levels of physical activity have been previously reported by most studies in the field to be associated with an increased risk of obesity, according to one review of the evidence [28]. Additionally, a recently published meta-analysis of 73 studies reported that, overall, there was a small but significant effect of physical activity levels on children's mental health [29]. Moreover, the Department for Health for England has recognised the importance of physical activity and has issued guidelines recommending 30-59 minutes of moderate to vigorous physical activity per day [30]. Thus, physical activity level is a potential confounding factor when investigating the association between obesity and mental health in childhood.

The Health Survey for England conducted in 2007 (HSE 2007) was designed to place a special emphasis on information related to childhood obesity and also included estimates of psychological adjustment in those under 16 years [31]. This data presented an opportunity to explore the cross-sectional relationship between excess adiposity and mental wellbeing in children and model any association in a more sophisticated way than has previously been reported. Thus, the study objectives were: to test whether a relationship between adjusted BMI and parental ratings of child emotional and behavioural health was observed; whether this potential relationship was independent of putative confounding variables and; the nature and strength of any association observed.

## Methods

### Ethics

As this project involved only secondary analysis of anonymised publically available data ethical approval was not required. Ethical approval for the original data collection was granted by the London Multi-Centre Research Ethics Committee.

### Participants

Data from the HSE 2007 was utilised. Information on under 16 year olds was obtained from two components of the survey. First, data on children living with adults were gathered as part of the stratified random 'core sample' of 7,200 households in England. Second, a 'child boost' component to the survey obtained information on children from a stratified random sample of 26,100 selected addresses [32]. In both cases, where more than two children resided at the address two children were randomly selected for interview. Consequently a total of 6,882 adults and 7,504 children were interviewed, with 1,727 children from the core sample and 5,777 from the boost. Those aged 13-16 were interviewed directly about health and lifestyle issues whilst this information was obtained via parents for younger participants. The full methodology of the HSE 2007 is detailed in the survey technical documentation and reports. In terms of sociodemographic characteristics the samples were representative at both a regional and national level [32]. For the purposes of this analysis only data from children aged 5-16 years was utilised; this is the age range for which the Strengths and Difficulties Questionnaire (SDQ) has been validated.

### Measures

Interviewers measured the weight and heights of children. These were first converted to BMIs (kg/m^2^) then to standardised BMI z-scores that were adjusted for age and gender using data obtained from the 1990 growth reference dataset [33]. Children were then classified as overweight or obese according to the International Obesity Task Force (IOTF) recommended cut-offs for standardised BMI [34].

Socioeconomic status was evaluated according to equivalised household income (total household income adjusted for the number of people dwelling there). Ethnicity was reported to interviewers and grouped into White/Black/Asian/Mixed and 'Chinese or other' ethnicities. Estimated time spent engaged in physical activity over the preceding week was also reported to the interviewer. Where reported activity levels were less than 30-59 minutes of moderate to vigorous physical activity per day over the last seven days the child was categorised as having activity levels likely to be significantly below the current Department of Health for England recommendations [30].

The parentally completed version of the Strengths and Difficulties Questionnaire (SDQ) was used to evaluate child psychological wellbeing [35]. The SDQ is traditionally divided into five subscales (Conduct Problems, Emotional Symptoms, Hyperactivity, Peer Problems and Prosocial Behaviour) according to the originally proposed factor structure. An overall estimate of psychological adjustment is derived from the summed scores of the former four of these five subscales (the total difficulties score). The SDQ has been validated against semi-structured diagnostic interviews in terms of the instruments ability to detect clinically significant behavioural or emotional disturbance. The parental version of the instrument has 62.1% sensitivity to detect any psychiatric disorder, 73.5% sensitivity to detect clinically significant conduct problems and 69.2% sensitivity to detect depression in children aged 5-10 years. For children aged 11-15 years these values are 59.4%, 77.3% and 61.1% respectively [36]. Thus, as might be expected, parental reports using the questionnaire are better at detecting behavioural rather than emotional problems. Despite this, it should be noted that the parental SDQ is better at detecting depression in children and adolescents than the self-report version of the instrument. A recent reanalysis of a large community-based sample of SDQ respondents suggests that in non-clinical (i.e. low-risk) populations a scoring system based on a three factor structure (internalising, externalising and prosocial behaviour) may be more appropriate [37]. This, more parsimonious, structure was reported to show the clearest and most consistent evidence of convergent and discriminant validity across informants and reliability with respect to the diagnosis of clinical disorder. Thus, using the broader internalising and externalising dimensions may therefore be more appropriate as predictor or dependent variables for epidemiological studies. For this reason, when evaluating emotional and behavioural symptoms, factor scores were utilised as the estimates for the internalising (emotional) and externalising (behavioural) latent variables respectively. Factor (rather than summed) scores were utilised in this case as in the present sample factor loadings were found not to be tau-equivalent (i.e. factor loadings significantly varied across items). However, normative data on this alternative SDQ structure is not yet available. Therefore for mental health screening purposes the recommended cut-off score of five or more for both Conduct Problems and Emotional Symptoms subscales of the SDQ was utilised [36]. Screening also usually utilises the SDQ 'impact score'. This reports whether the parent considers the child's functioning has been affected by any reported symptoms. As the impact supplement was not included in interview schedule for the HSE 2007 screening thresholds were defined on the basis of subscale total scores only, computed on the basis of the algorithm provided by the questionnaire authors on the SDQ website [38].

### Statistical Analysis

As clustering occurred due to second stage sampling procedures a multilevel approach to model evaluation was utilised to allow for the non-independence of observations from children nested within the same home. Thus, a random intercept with covariates model was used to explore the relationship between the dependent (reported psychological adjustment) and predictor variables. Sampling weights can potentially be employed in the multilevel analysis of complex survey data but both cluster and individual level weights must be rescaled [39]. As cluster level probability sampling weights were not available for children in the child boost sample this strategy could not be used. When investigating potential cross-level effects, random coefficients for the regression slopes between obesity and internalising/externalising factor scores were also introduced. Household income was therefore treated as a level two variable whilst other observations were on the child level (level one). Dummy variables were created for categorical items used in regression-based analyses. Continuous explanatory variables were mean-centred. In order to examine the likelihood of a child exceeding the SDQ screening threshold score for a potentially clinically significant emotional or behavioural disorder a multilevel logistic regression was performed. Thirty quadrature points were specified to ensure accurate estimates.

All analyses were performed using Stata SE version 11 [40], with the exception of the investigation of cross-level interaction and derivation of factor scores which utilised Mplus version 6 [41]. Factor scores were derived via a Confirmatory Factor Analysis (CFA) performed using Robust Weighted Least Squares as the estimation method to allow for the ordinal nature of the SDQ ratings.

## Results

Sixty-six percent of all eligible households in the general sample and 75% of those eligible for the child boost sample participated in the HSE 2007. Within cooperating households 99% of children participated in the survey [18]. Information from 5,779 children in the target 5-16 years age range was available; 1,193 obtained via the core and 4,586 from the child boost survey sample. Of these 3,955 (89%) had both a validated Body Mass Index (BMI) and a completed parental SDQ available. Of these 3,679 (93%) had no missing SDQ responses and 3,898 (99%) had only one or no missing responses. Thus, the final analysis utilised data from these 3,898 children.

There was no significant difference in terms of household income (p=.9), age (p=.4), gender (p=.4) or adjusted BMI (p=.9) between those that had and had not parental completed SDQs available. The mean standardised BMI (Z score) was .59 (sd 1.2). The range of standardised BMIs was from 9.68 standard deviations below the mean to 6.14 standard deviations above the mean, with the interquartile range for z scores being from -.12 to 1.35. Consequently 991 (25%) of the final sample were classified as overweight/at-risk of obesity (85^th ^- 95^th ^centile based on IOTF normative data) and 377 (9%) as obese ( > 95^th ^centile). Overall, girls were not more likely to be classified as obese compared to boys (χ^2 ^= 1.30, p=.3). However, if the sample was stratified by age then it was observed that those under 10 years that were obese were more likely to be female (χ^2 ^= 4.72, p=.03). No such sex difference was observed for those over 10 years of age (χ^2^=.06, p=.8).

### Sociodemographic characteristics

For those participants aged 5-16 years with a valid BMI and completed SDQ the mean age was 10.1 years (sd 3.1) and 51% (2,017) were male. Average equivalised household income was £25,644/year and the mean daily physical activity levels reported were 89 minutes/day (sd 88 minutes). In terms of ethnicity 3,392 (85.8%) of the sample were classified as White, 258 (6.5%) as Asian, 137 (3.5%) as Black, 135 (3.4%) as Mixed and 31 (.8%) as Chinese/Other. Ethnicity was not reported in three cases.

### Univariate Analysis

**Figure 1:**
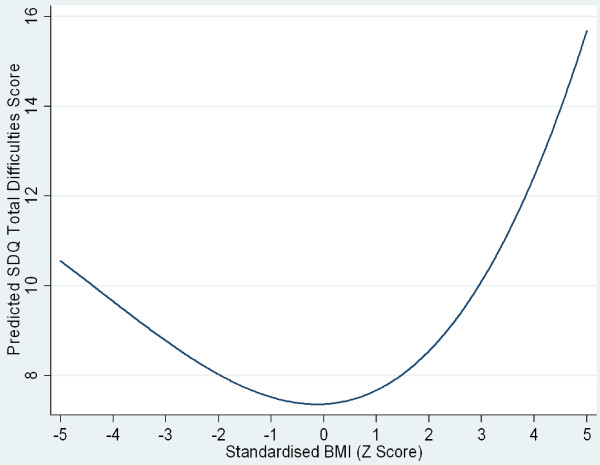
**Relationship between adjusted BMI and psychological wellbeing (parentally reported Strengths and Difficulties Questionnaire Total Score) predicted from regression model, adjusted only for non-independence of observations for children in the same families**.

A univariate analysis was performed to explore the relationship between parentally reported psychological adjustment and obesity and also to identify any potential confounding/mediating variables. Both mean total SDQ score (as a marker of overall psychological adjustment) and the internalising (emotional) and externalising (behavioural) symptoms factor scores were significantly higher in children classified as obese but not overweight, according to the IOTF recommended cut-offs (see Table 1). In order to explore the crude association between mental wellbeing and weight, total SDQ core was regressed on age and gender adjusted BMI. A random intercept term was introduced to allow for the non-independence of children within the same families. As adjusted BMI was in the form of a Z score, a constant was added so that all values were positive, allowing the addition of quadratic terms to the model. Indeed, the addition of quadratic and cubic terms, though not higher polynomials, increased the fit of the modelled association between adjusted BMI and SDQ total score, reflecting a curvilinear relationship between weight and psychological wellbeing. This modelled relationship is depicted in Figure 1 for the SDQ total scores. However, the overall amount of variance in the SDQ total scores explained by BMI was small at 1.9% (R^2 ^for within family effects=.005, between effects=.024, overall R^2^=.019).

**Table 1:** Parentally reported mean Strengths and Difficulties Questionnaire (SDQ) Total scores and (standardised) Factor Scores for "Internalising" and "Externalising" factors by International Obesity Taskforce (IOTF) classification

IOTF Classification Status	Mean SDQ Total (sd)	Mean Internalising Factor Score (sd)	Mean Externalising Factor Score (sd)	N (%) Emotional Disorder Screen Positive	N (%) Conduct Disorder Screen Positive
Obese (n = 305)	10.18 (6.1)*	.17 (.4)*	.21 (.6)*	53 (17%)*	48 (16%)**
Overweight (n = 760)	8.07 (5.4)	.06 (.4)	.02 (.6)	84 (11%)	84 (11%)
Normal weight (n = 2,704)	7.57 (5.4)	-.01(.4)	.00 (.6)	226 (8%)	275 (11%)
Underweight (n = 192)	7.59 (5.1)	.01 (.4)	.00 (.6)	16 (8%)	14 (7%)

Note: *Difference between "Obese" and other groups significant at p < .001 level; **Difference between "Obese" and other groups significant at p < .01 level

Increasing child age was significantly associated with increasing total SDQ internalising score and a significant trend to increased adjusted BMI. No gender difference in internalising factor scores were observed. Equivalised household income was associated with both increased BMI and SDQ internalising factor scores. In terms of ethnicity, those reporting Asian ethnicity had higher internalising symptom scores but lower BMIs and household incomes, on average, when compared to non-Asian participants. When treated as a continuous variable reported weekly physical activity levels were observed to have a quadratic relationship with internalising symptoms scores. When physical activity was dichotomised as below/above recommended levels for England low activity status was associated with higher internalising symptom scores compared to those who reported exceeding the recommended levels of physical activity. Thus, low physical activity levels, income, age and BMI/obesity status were entered into the multilevel multiple regression model predicting internalising symptoms factor score as potential confounding/mediating variables.

In terms of externalising symptoms: obesity was associated with higher scores and a similar curvilinear relationship with adjusted BMI was observed (not shown); no associations with ethnicity were observed. There was no association between low physical activity status and externalising factor scores. Girls had lower mean externalising scores than boys and slightly lower adjusted BMIs. Increasing income was associated with both lower externalising behaviour scores and adjusted BMI. Increasing age was correlated with higher BMI but lower externalising scores. Consequently, only income and gender were entered into the multivariate regression model exploring the association between reported externalising behaviours and obesity.

### Multilevel modelling

Using adjusted BMI as a continuous measure, the cubic relationship with internalising symptoms factor scores was reduced but remained statistically significant (p=.02) once the effects of age, low physical activity levels, equivalised household income and non-independence of observations from children nested in the same household were adjusted for. Likewise the cubic association between adjusted BMI and externalising factor scores was slightly reduced in magnitude but remained statistically significant (p=.009) once the effects of gender and household income were controlled for (full results not shown).

Using a dichotomous approach to BMI (obese vs non-obese) all variables included in the model predicting internalising factor scores, except age, were significantly and independently associated with internalising factor scores (see Table 2). Likewise, all the explanatory variables in the model predicting externalising factor scores were significant at the p < .001 level (see Table 2). The results of a multilevel logistic regression showed that the odds ratio (OR) of exceeding the SDQ screening threshold for an emotional disorder was 2.13 (95% CI 1.39 to 3.26) for an obese compared to a non-obese child, once the effects of potential confounders were adjusted for. However, using the screening cut-off for the conduct problems subscale, it was observed that the association between obesity and exceeding the screening threshold for conduct problems was only of borderline statistical significance once the effects of income and gender were controlled for (OR 1.58, 95% CI 1.00 to 2.50)(see Table 3). Consequently an income/gender interaction term was introduced into the model. However this was not a significant predictor of 'screen positive' conduct problems (OR .94, 95% CI .78 to 1.13, p=.5).

**Table 2:** Findings from a multiple regression using a Random Intercept with Covariates model to allow for the nesting of children within families

Internalising Factor scores		
Variable	Coefficient	95% CI
Obesity	.14	.09 to .18*
Age	.00	.00 to .01
Low Activity Levels	.07	.03 to .10*
Equivalised Family Income (per £10k)	-.03	-.04 to -.02*
Variance across families	.04	-
Residual individual variance	.08	-
ICC	.34	-

Externalising Factor scores		
Variable	Coefficient	95% CI

Obesity	.19	.11 to .26*
Female gender	-.17	-.21 to -.13*
Equivalised Family Income (per £10k)	-.05	-.05 to -.04*
Variance across families	.06	-
Residual individual variance	.25	-
ICC	.20	-

Note: CI = Confidence Intervals, ICC = Intraclass correlation for Factor Scores for children within the same household.*All sig at p < .001

**Table 3:** Multilevel logistic regression showing the odds ratios (and 95% confidence intervals) of exceeding the SDQ screening threshold for an emotional or behavioural disorder, unadjusted and by obesity status, age, equivalised family income and reported physical activity levels (low vs above recommended levels)

Emotional Disorder Screen Positive				
Variable	Unadjusted ORs (95% Confidence Intervals)	p value	Adjusted ORs (95% Confidence Intervals)	p value
Obesity	2.33 (1.55 to 3.50)	< .001	2.13 (1.39 to 3.26)	.001
Low Activity Level	1.80 (1.33 to 2.45)	< .001	1.62 (1.16 to 2.27)	.005
Family Income (per 10k)	.80 (.73 to .87)	< .001	.80 (.73 to .87)	< .001
Conduct Disorder Screen Positive				
Variable	Unadjusted ORs (95% Confidence Intervals)	p value	Adjusted ORs (95% Confidence Intervals)	p value

Obesity	1.74 (1.13 to 2.67)	.01	1.58 (1.00 to 2.50)	.05
Female Gender	.58 (.45 to .75)	< .001	.60 (.45 to .79)	< .001
Family Income (per 10k)	.74 (.68 to .81)	< .001	.76 (.69 to .83)	< .001

A random slope model was used to investigate cross-level interaction; in this case whether household income modified the relationship between obesity and reported emotional or behavioural symptoms. There was no evidence of a moderating effect of household income on the relationship between obesity and either internalising or externalising symptom factor scores (β=.01, p = 0.4 and β=.00, p=.99 respectively).

Residual diagnostics were performed for the multilevel multivariate models used in the analysis via plots of residual values for both the fixed and random effects. These indicated that the residuals were normally distributed. In order to check for endogeneity a Hausman test was conducted, which did not indicate significant model misspecification via endogenous within household effects (p=.5).

## Discussion

In this sample, childhood obesity was significantly negatively associated with parental reports of psychological adjustment. It is important to stress that, overall, adjusted BMI accounted for only a very small fraction of the variance in reported psychological health. This indicated that childhood BMI accounts for an almost negligible amount of the variance in parentally reported child psychological adjustment across the entire adjusted weight range. Nevertheless, the tentatively modelled curvilinear relationship between weight/reported exercise and mental health strongly suggested the presence of threshold effects. These were indeed evidenced by the results of the analysis once both BMI and SDQ scores were dichotomised. In particular the risk of an emotional disorder was independently increased by obesity. Whilst higher externalising symptom factor scores were associated with obesity, the risk of exceeding the screening thresholds for Conduct Disorder were only weakly increased, once adjusted for the influence of potentially confounding variables. This apparent discrepancy is most likely to be due to the externalising factor including items from both the SDQ peer problems and hyperactivity symptoms subscales as well as the five items that make up the original Conduct Problems subscale. Thus the externalising factor represented a broader construct than that captured by the traditionally used SDQ Conduct Problems subscale. Indeed, it may be the potential difficulties in peer relationships that the externalising factor scores are detecting in children classified as obese. It is not clear why there is a trend for poorer adjustment at lower standardised BMIs. However, feeding and eating difficulties, resulting in an underweight child, may be associated with a number of psychiatric disorders, including autism spectrum disorders [42] and, by definition, anorexia nervosa. Moreover, low weight and failure-to-thrive may also be a marker of an adverse home environment, resulting in an increased risk of psychological problems [43].

### Comparison with Previous Findings

This sample of children had, on average, higher BMIs than those used to derive normative values in 1990 [33] reflecting the overall trend for increased obesity rates over the last two decades. As the IOTF recommended cut-offs for overweight and obesity were employed the rates presently reported will be lower than those already described in the HSE 2007 report, which utilised normative data from the UK only [31]. Our observation of higher rates of obesity in girls compared to boys under 10 years is a trend that has been observed in health survey data since the mid 1990s [44].

Our finding of an independent association between obesity and internalising (emotional) difficulties is echoed by findings from a smaller, mainly non-White multiethnic sample of 11-14 year olds from East London. In the survey by Viner and colleagues, 17% of those of White British ethnicity (N = 267) who were classified as obese scored above screening threshold for self-reported SDQ total difficulties compared to 9% of ideal weight children of the same ethnic group [19]. Overall differences in SDQ total difficulties scores remained significant even after controlling for gender, age and socioeconomic status. A significant, independent association with depression and chronic obesity was observed in boys (but not girls) in an all-white sample of 9-16 year olds (N = 991) drawn from the US-based Great Smoky Mountains study. The authors reported that boys with depression were 1.7 times more likely to be chronically obese than non-depressed boys after controlling for SES and age [21].

However, the above findings stand in contrast to those reported by several previous studies; one Dutch survey of 614 children aged 13-14 reported a statistically significant relationship between obesity and only the peer problems/prosocial behaviour subscale scores of the self-report version of the SDQ, once age, gender and educational status had been adjusted for [45]. A separate survey of 4,320 London-based school students age 11-12 years utilised the self-report SDQ and reported only a small ( < 1 point on the SDQ) though statistically significant (p=.01) trend for the SDQ Emotional Symptoms subscale score to be raised in obese and overweight children compared to ideal weight peers [19]. The authors attempted to control for the effect of potential confounding variables by sub-group analysis according to ethnicity, socioeconomic group (based on Townsend scores) and gender. As in our study, the authors concluded that there was no evidence that socioeconomic status was a moderating variable, although a sub-group analysis may have lacked power to detect a difference, should it have existed. Ethnicity and gender were highlighted as potential moderating factors with the closest association between obesity status SDQ total scores being observed in the subgroup of girls of white ethnicity (mean score of 12.1 [obese] vs 13.4 [ideal weight]). The lack of association between overweight, as opposed to obesity, and poor mental health observed in our cohort of British children echo the findings from a community-based survey of 2,341 French children aged 6-11 years [46]. This latter study found no association with Conduct Problems or Emotional Symptoms SDQ scores and weight exceeding the 85^th ^centile once sociodemographic and lifestyle (including physical activity levels) were adjusted for. These findings, along with the curvilinear relationship between adjusted BMI and emotional symptoms reported by the present study, strongly suggest the presence of a threshold effect of childhood BMI on psychological wellbeing. Thus, we would hypothesise that the risk of significant emotional problems would rapidly increase in children with BMI z-scores exceeding approximately 2.0 (i.e. exceeding the 97^th ^centile). As with existing studies, BMI explained only around 2% of the variance in SDQ scores. Nevertheless, taking a categorical approach, obesity would appear to be associated with a clinically significant risk of poor psychological adjustment, at least in terms of emotional difficulties due to the potential threshold effects outlined above. In addition, it must be noted that the SDQ was developed as a screen for mental health problems in young people and the instrument may be less useful as a metric of wellbeing. However, the variation in published findings are unlikely to be wholly explained by the different measures employed. Rather, there may be genuine differences in the relationship between childhood obesity and wellbeing as a result of both cultural and cohort effects which require further exploration. The choice of potential mediating/confounding variables may also shape the final results.

This is not the first study to observe some relationship between BMI and externalising problems in children. Indeed, findings from both a British cohort reported higher rates of externalising problems in obese boys aged 3-5 years [22]. Moreover, a study of a North American cohort of children of both sexes reported that children with externalising behaviour problems at 2 years old had significantly higher BMIs when followed-up at age 12 years [47]. However, overall, the association of behavioural problems with obesity seems less consistent than that with emotional difficulties, as echoed by the present findings.

In the present study we did not observe a difference in internalising factor scores according to gender. Given the previously documented excess of depression and anxiety in adolescent females this was initially surprising. However, in the present study the average age of the study sample was only about 10 years and the gender difference in emotional problems may only become apparent in later teenage years. For example, depression is twice as common in adolescent girls compared to boys but this difference is only observed by the age of 15 years [48]. Moreover, higher rates of comorbidity between internalising and externalising difficulties have been reported in pre-pubescent boys [49] and this also may have contributed to a lack of an observed gender difference.

### Study Strengths and Limitations

This was a relatively complete and representative national sample of children where the effects of a number of key sociodemographic variables were able to be controlled for. Moreover, the use of multilevel modelling appropriately adjusted the standard errors of the estimates for the non-independence of observations from children nested within households. However, although there were a very large number of clusters the average number of children nested within families was small at 1.4. Indeed, given this average cluster size and the intraclass correlations for observations nested within families the design effects were relatively small, and the curve in Figure 1 would not appear very different were these not controlled for by the introduction of a random intercept to the model. Nevertheless, given the clearly hierarchical nature of the data and the risk of dependency amongst residuals from observations within each cluster we felt the use of multilevel, rather than single level, modelling was justified. Moreover, this approach provided an opportunity to explore, albeit tentatively, within family effects and cross-level interaction. However, when considering the power of multilevel modelling studies both cluster number and size, as well as the parameters being estimated must be taken into account. When estimating parameters associated with fixed-effects (e.g. the effect of obesity status externalising factor scores) the number of clusters are of prime importance- where fewer than 50 clusters exist parameter estimates may be biased downwards [50]. Therefore it can be assumed that any fixed effects were estimated accurately. However, in this analysis we also introduced a random slope parameter in order to investigate the possibility of cross-level interaction. Again, cluster size is of secondary importance to the number of clusters with a recommendation of at least 100 groups with around 10 individuals in each group [51]. However, in our study average cluster size was considerably lower than this, although the number of clusters was very large. Therefore the parameters associated with potential cross-level interactions may be relatively poorly estimated and we may not have detected a significant effect where one existed. This is a potential limitation of the present study. Nevertheless, our findings were in keeping with that of Drukker *et al. *[45] who also reported that SES did not appear to be a moderating factor. However, neither the present or these latter findings can be taken as definitive evidence of this as both studies may be subject to low power.

Ideally, more detailed biometrics would have been utilised to derive obesity status. However, the IOTF recommended cut-offs correlate to a moderate to high degree with more sophisticated methods to estimate adiposity [52]. Whilst valid BMIs were obtained, self-reported physical activity levels may be less reliable than more objective based estimates, such as those based on accelerometry or heart rate, although moderate levels of correlation are generally reported [53]. No information on pubertal status was available in this sample. The relatively small numbers of non-white ethnic groups within this survey, whilst reflecting the general population from which the sample was drawn, makes it difficult to draw firm conclusions about ethnic differences. Probably the most significant limitation in this survey was that data on psychological wellbeing was restricted to the parentally reported SDQ, in the absence of the SDQ impact supplement. The use of SDQ internalising and externalising factor scores as the main outcome measure may have been more appropriate than using SDQ subscale scores consisting of only five items each. Moreover, parentally reported SDQ scores may be more sensitive to emotional disturbance than the self-report version of this instrument in 11-15 year olds [36]. Indeed, the use of totalled subscale scores in previous studies could partly explain the failure to report firm associations between obesity and emotional problems in young people. The SDQ is widely used and well validated, but the addition of self-report versions for those children over ten years would have resulted in increased sensitivity for the screening for potentially clinically significant disorders [35,36]. The exclusion of the impact supplement from the survey pack may have reduced the reliability of the screening thresholds for conduct and emotional disorders as defined by the respective SDQ subscales. Despite this, the relative risks may have remained relatively unchanged as the decreased accuracy would apply to both obese and non-obese children. In addition, we did not have any detailed information of family environment available, although we felt it was important to include family level economic status as this is known to be a risk factor for both childhood obesity [54] and certain psychological problems [55].

### Directions for Future Research

The conflicting findings from previously published research suggest that further datasets containing relevant measures of wellbeing and biometrics should be utilised in replicating the present analyses. However, in order to model hypothesised underlying mechanisms driving the association further longitudinal data are required. A number of ongoing studies of health and development are potential sources of such information, though it may be that new studies based in mixed qualitative/quantitative methodologies would be more effective in exploring this area and contextualising classes of observed trajectories. There are some indications that in adults poor mental health (and in particular, depression) may precede obesity [16]. There is little longitudinal research published regarding under 18s but the available evidence suggests this predominant direction of causality may also apply to children and adolescents. One US based longitudinal study involving 9,374 adolescents reported no association between obesity and depression at initial assessment. In contrast, at one year follow-up, depression significantly predicted onset of obesity (OR 2.05; 95% CI 1.04 to 4.06) independent of self-esteem ratings, conduct problems, socioeconomic status, gender and parental obesity [56]. A separate cohort study also suggested that childhood depression was a risk factor for obesity in adulthood, at least for women [44]. 'Temperamental Difficulties' were also noted to predict weight gain in a cohort of 138 North American children aged between 4 and 9 [57]. From these scant studies a tentative model could be proposed whereby temperament (largely hereditary in nature), interacting with early environment gives rise to a tendency to dysphoric mood and low self-esteem that increases the risk of over-eating. The reasons for the non-linearity of the relationship between BMI and psychological adjustment require further exploration. It may be that socio-cultural factors are the predominant influence, with children who obviously exceed the normative range of adiposity being at an exponentially increasing risk of adverse experiences, such as peer rejection.

## Conclusions

In this large and nationally representative cohort there was evidence of a threshold effect of obesity on reported mental wellbeing in children. This association remained even after the effects of potential confounding factors were controlled for.

There has been some debate regarding whether public health initiatives which address obesity should target diet or physical activity [58]. Our analysis indicated that the impact of obesity on psychological health was largely independent of reported physical activity levels. The curvilinear relationships noted between the lifestyle related variables (reported physical activity and BMI) and psychological wellbeing and potential threshold effects support the use of centralised recommendations, such as those produced by the Department of Health for England and continued efforts should be made to implement these [30]. The present findings suggest that those children exceeding the BMI threshold for obesity are more likely to be affected by emotional disorders. Given our current knowledge of the long-term outcomes of both childhood mental health problems as well as the recognised complications of chronic obesity this has implications for the long-term health and social care burdens in the developed world. Policy makers are likely to continue considering universal-level public health interventions such as social marketing campaigns linked to obesity. However, it may be that interventions targeting individuals may also prove to be cost-effective, given the well-recognised challenges to health-related behaviour change. For children, family-based interventions may be required in order to improve both behaviours related to good psychosocial as well as physical functioning [59]. A variety of approaches are also available that may prove invaluable in encouraging children towards healthier behaviours. For example, Behavioural Activation is a brief psychotherapy that has been successfully piloted in working-age adults with comorbid depression and obesity [60]. Given the direct and indirect costs of obesity to individuals and society it is likely that even relatively expensive, but effective, interventions would pay for themselves over the medium to long-term.

## Competing interests

The authors declare that they have no competing interests.

## Authors' contributions

PAT led on conceptualisation, data analysis and writing of the report. BA performed much of the literature reviewing and contributed to the writing of the report. HJM contributed to appraising the content and the writing of the report. CDS contributed to the supervision and conceptualisation of the project and the writing of the report.

All authors read and approved the final manuscript.

## Authors' Information

PAT is an academic child and adolescent psychiatrist with an interest in epidemiology and applied statistical modelling. BA is developmental psychologist with an interest in mental health problems of childhood. HJM is a post doctoral research associate in the Obesity Related Behaviours Research Group at Durham University. CDS is the director of the Obesity Related Behaviours Research Group and Professor of Human Nutrition at Durham University.
